# Implicit Target Substitution and Sequencing for Lexical Tone Production in Chinese: An fMRI Study

**DOI:** 10.1371/journal.pone.0083126

**Published:** 2014-01-10

**Authors:** Hui-Chuan Chang, Hsin-Ju Lee, Ovid J. L. Tzeng, Wen-Jui Kuo

**Affiliations:** 1 Institute of Neuroscience, National Yang-Ming University, Taipei, Taiwan; 2 Institute of Linguistics, Academia Sinica, Taipei, Taiwan; 3 Brain Research Center, National Yang-Ming University, Taipei, Taiwan; University Children's Hospital Tuebingen, Germany

## Abstract

In this study, we examine the neural substrates underlying Tone 3 sandhi and tone sequencing in Mandarin Chinese using fMRI. Tone 3 sandhi is traditionally described as the substitution of Tone 3 with Tone 2 when followed by another Tone 3 (i.e., 33→23). According to current speech production models, target substitution is expected to engage the posterior inferior frontal gyrus. Since Tone 3 sandhi is, to some extent, independent of segments, which makes it more similar to singing, right-lateralized activation in this region was predicted. As for tone sequencing, based on studies in sequencing, we expected the involvement of the supplementary motor area. In the experiments, participants were asked to produce twelve four-syllable sequences with the same tone assignment (the repeated sequences) or a different tone assignment (the mixed sequences). We found right-lateralized posterior inferior frontal gyrus activation for the sequence 3333 (Tone 3 sandhi) and left-lateralized activation in the supplementary motor area for the mixed sequences (tone sequencing). We proposed that tones and segments could be processed in parallel in the left and right hemispheres, but their integration, or the product of their integration, is hosted in the left hemisphere.

## Introduction

Lateralization of language network to the left hemisphere [1]–[4] is often thought to be domain-specific [5], [6]. However, it could also be the case that regions serving domain-general functions—e.g., the processing of physical properties in speech input and output—in the left hemisphere are recruited for language processing [7]. Segments, including vowels and consonants, are phonological units in all languages. In contrast, pitch is used in tone languages only to distinguish words. Compared to phonological segments, perception of non-speech pitch is known to be right-lateralized [8]–[10], probably reflecting the processing of its physical properties, such as longer duration (approximately 150–250 ms for tone and 20–40 ms for segments) [11], [12] and richer spectral information [10], [13]. It has even been argued that language is lateralized because of its interaction with the auditory and motor systems during learning and on-line monitoring [14]. To what extent does lateralization of language depend on the physical properties of speech input/output? Understanding of how our brain processes lexical tones should be able to shed some light on the answer of this question.

There are four lexical tones in Mandarin Chinese. Each syllable bears one tone. The same syllable can indicate different meanings by carrying different tones. Imaging studies on tone perception have shown that, in comparison to segments, the processing of lexical tones elicit more activation in the right hemisphere [15], [16]. However, studies have also shown that, for native Chinese speakers [17]–[20] and trained English speakers [21], tone perception is more left-lateralized than it is for untrained English speakers. Taken together, tone processing needs the expertise of both the right hemisphere for auditory analysis and that of the left hemisphere for linguistic processing [16], [22]. For the involvement of the left hemisphere, it was observed only in those who learned tonal languages [17]–[21]. Since little semantic, syntactic, and lexical processing was involved in these experiments [23], the “higher linguistic process” could be purely phonological. One candidate for this process is the categorization of pitch, which is supported by perception studies showing that cross-category variation elicits more activation in the left hemisphere than the within-category variation [24], [25]. The other candidate process is the integration of tone and segment. In this paper, we would like to examine the later argument with the Tone 3 sandhi in Mandarin Chinese.

Tone 3 sandhi is traditionally described as the substitution of Tone 3 with Tone 2 when followed by another Tone 3 [26]—i.e., tone sequence 33 is pronounced as 23. It implies that Tone 3 sandhi changes the target of articulation rather than the way of its implementation, and this change is independent of segments [28]. If the hypothesis that left lateralization of tone processing of experienced speakers reflects improved integration of tones and segments, tone processing itself that is independent of segments—e.g., Tone 3 sandhi—is not necessarily left-lateralized.

For speech production, while the articulatory target is relatively invariant, its implementation is often found to be modified for ease of articulation [27]–[31]—e.g., coarticulation [27]–[31]. An example of tone coarticulation is the assimilation of a tone's onset fundamental frequency (F0) to the offset of the preceding tone [28]. Evidence supporting the view that Tone 3 sandhi changes the tone target for articulation, rather than the way of its implementation, is enumerated as follows. First, it is hard to discriminate sandhi Tone 3 from Tone 2, either perceptually [32], [33] or acoustically [33]–[35]. Second, compared with the contextual factors to increase ease of articulation, Tone 3 sandhi appears much later and less accurate along development [36]. Third, little evidence supports that the application of Tone 3 sandhi increases ease of articulation [37], [38].

Distinction between the invariant target and its implementation is a common feature of current speech production models [39]–[44]. In Levelt et al.'s model [45], an invariant target is a segment. After retrieval, segments are syllabified and stress is assigned in the syllabification/prosodification stage. Then, the outputs are implemented in the phonetic stage. Drawing an analogy between tone and segment, Tone 3 sandhi should be applied after the retrieval of the invariant tone target and before the implementation stage—i.e., in the syllabification/prosodificatoin stage. A meta-analysis study indicated that the syllabification/prosodification stage is most likely to be hosted in the left posterior inferior frontal gyrus (IFG) [1]. In the hierarchical state feedback control model (HSFC), two processing loops—the auditory-Spt (a region in the left posterior Sylvian fissure at the parietal-temporal boundary)-BA44, and the somatosensory-cerebellum-motor loops—are hierarchically organized [44]. While targets with invariant acoustic features reside in the higher, auditory control loop—e.g., syllables—targets with variant acoustic features reside in the lower, somatosensory loop—e.g., segments. For each target type, a motor program and an auditory target are activated in parallel, and whether they match with each other is checked through internal feedback signaling. For lexical tones, they can be reliably distinguished by their acoustic feature—i.e., the fundamental frequency. The motor program for a tone is likely to reside in BA44. In brief, both models predicted involvement of the left posterior IFG for Tone 3 sandhi processing.

Since most current speech production models pay little attention to tone processing, we can apply these models only by analogy between tone and segment or syllable. However, when taking the physical properties of tone into consideration, the predicted posterior IFG activation is not necessarily left-lateralized because, physically, tone production is similar to singing, and singing is right-lateralized as pitch perception. Studies that directly compare singing and speaking have shown opposite hemispheric lateralization in IFG [46], superior temporal gyrus (STG) [46], [47], and insula [48], [49]. For singing, the right hemispheric parts of these areas are suggested to play a similar role as their left hemispheric counterparts in speaking [46], [50]. A recent study shows that the volume of the right ventral arcuate fasciculus connecting right IFG and right STG is positively correlated with the performance in pitch-based artificial grammar learning [51]. The damaged right ventral arcuate fasciculus also has been shown to result in impaired processing of both non-speech pitch [8], [52], [53] and lexical tone [54]. If the influence of physical properties of speech input/output on lateralization is not limited to early auditory analysis, the right-lateralized singing network should participate in tone production.

There are few studies on tone production. Using the adaptation paradigm, Liu et al. [55] compared the production of vowels and tones in monosyllables. They found that although both vowels and tone show left hemisphere dominance, the activations in the IFG, insula, and STG were less left-lateralized for tone changes. We hypothesize that tone processing is right-lateralized before its integration with segments. Tone 3 sandhi requires segment-independent tone target processing. Therefore, we predict right-lateralized activation in the posterior IFG for Tone 3 sandhi.

In addition to Tone 3 sandhi, we are also interested in tone sequencing. The mechanism of sequencing has been studied with sequences of syllables and finger movements. Using single-cell recording on monkeys, Shima and Tanji [56] found that cells in the major part of the supplementary motor area (SMA) respond selectively to the initiation of movement sequences and cells in pre-SMA respond selectively to transitions between certain movement pairs in the sequences. Human imaging studies show that mixed-movement sequences increase brain activation in the contralateral SMA, pre-SMA, contralateral premotor areas, and bilateral inferior parietal lobule [57], [58]. Similarly, the same areas are found to be engaged in syllable sequencing [59]. We predict that similar regions are recruited by tone sequencing, especially SMA. Assuming that tone target is processed in the right hemisphere and the composite of tone and segments is processed in the left hemisphere, the lateralization of SMA could clarify the unit of sequencing during tone production.

In this study, behavioral and fMRI data were collected during production of twelve tone sequences. The brain regions engaged in Tone 3 sandhi were expected to be revealed by sequence 3333 and brain regions engaged in sequencing were expected to be revealed by sequences of mixed tone (e.g., 2413). We hypothesized that segments and tones are processed in the left and right hemisphere respectively, while their integration, or the product of their integration, is processed in the left hemisphere. Because of its independence of segments, right-lateralized activation in the posterior IFG for Tone 3 sandhi was specifically expected. We also expected that the lateralization pattern of tone sequencing could help resolve the sequencing unit of Chinese.

## Methods

### Ethics Statement

Written consent was obtained before MR scanning, with the protocol approved by the Institutional Review Board of National Yang-Ming University.

### Participants

Fifteen college students were included in the behavioral experiment. Twenty-one college students were recruited for the fMRI experiment. All were right-handed, native Taiwanese Mandarin speakers, with no history of neurological disorders and normal or corrected-to-normal vision. Handedness of the participants was verified using the Edinburgh Inventory [60].

### Materials and procedures

**Figure 1:**
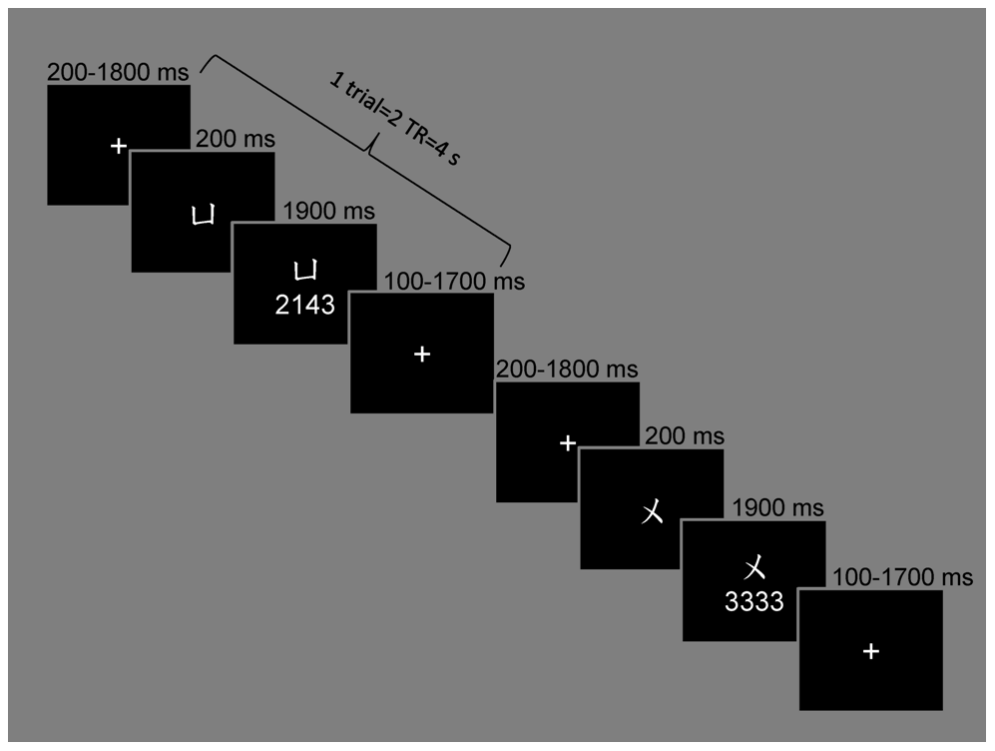
Examples of trials with mixed and repeated sequences. Left: a mixed tone sequence carried by the vowel /y/. Right: repeated sequence 3333, carried by the vowel /u/.

Forty-eight stimuli were created by combining four vowels and 12 tone sequences. There were 12 tone sequences in total: four repeated (1111, 2222, 3333, and 4444) and eight mixed (1234, 1324, 2143, 2413, 3142, 3412, 4231, and 4321). For the behavioral experiment, except the four-syllable sequences, 16 monosyllable stimuli were created by combining the vowels with the four tones. They were visually denoted as number sequences in the experiment (Figure 1). Four vowels—/a/, /i/, /u/, and /y/—were visually denoted as 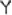, 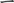, 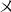, and 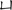 according to the phonetic noting system (*zhuyin fuhao*) used in Taiwan.

The behavioral experiment was conducted in a soundproof room for about an hour. There were two sessions in this experiment. The first session included 128 trials. Each of the 16 monosyllable stimuli was repeated eight times. The second session included 384 trials. Each of the 48 sequences was repeated eight times. In each trial, after a fixation of 500 ms, a vowel was presented alone for 200 ms. Then the tone appeared underneath the vowel for another 2,000 ms, followed by a blank for 1,000 ms in the first session or 2,000 ms in the second session. Erroneous responses were coded by the experimenter. Speech sounds were taped and digitized into 16-bit sounds with a sampling rate of 11 kHz.

In the fMRI experiment, for each participant, 240 trials and 480 images were acquired—two images per trial. For each trial, after a jitter period of 200–1,800 ms, in which a fixation cross was presented, the vowel was presented alone for 200 ms. Then the tone sequence appeared underneath the vowel for another 1,900 ms, followed by fixation until the next trial (Figure 1). The participants were asked to produce four syllables by repeating the vowel four times with the tones in sequence. Each of the 48 stimuli was repeated four times during the MR scanning, with 64 trials conducted for the repeated condition and 128 trials for the mixed condition, which were presented in random order. In order to effectively detect BOLD changes in response to the sequences presented, 48 null trials were included.

### MRI acquisition

The MR scanning was performed using a 3T MRI (Tim Trio, Siemens, Erlangen, Germany) interfaced with a 32-channel phased-array head coil. A T2*-weighted gradient-echo echo planar imaging (EPI) sequence was used for fMRI scanning, with slice thickness = 3.4 mm, in-plane resolution (64×64) = 3.44×3.44 mm, and TR/TE/θ = 2000 ms/30 ms/90°. Thirty-three axial slices were acquired to cover the whole brain. The anatomical, T1-weighted high-resolution image (1×1×1 mm) was acquired using a standard MPRAGE sequence (TR/TE/TI = 2530/3.49/1100 ms; flip angle = 7°). The total duration of the fMRI experiment was about one hour.

### MRI data analysis

A two-level analysis was implemented using SPM8. First, functional images were corrected for slice timing, head motion, normalized to the standard MNI brain space, and spatially smoothed with an isotropic Gaussian filter (8 mm full width at half maximum). Each individual participant's data was then modeled by six movement parameters and 12 regressors corresponding to the 12 tone sequences. The 12 regressors were obtained by convolving the impulse response with the canonical SPM hemodynamic response function and its time derivative [61]. Contrast images for each of the 12 regressors in the first level analysis were submitted to a second-level model with one regressor for each of the 12 sequences and one regressor for each participant. Repeated sequences (1111, 2222, and 4444) other than 3333 were taken as baseline. Contrasts of 3333 vs. baseline and mixed sequences vs. baseline were set to test effects of interest. The statistic threshold was set at p = 0.001, corrected at the cluster level (FDR p<0.05). Activation peaks within clusters were located using the Mascoi-toolbox for SPM [62] and labeled using Talairach Demon software [63] and xjView (http://www.alivelearn.net/xjview).

We calculated the lateralization index (LI) in regions showing significant effects in the two contrasts of interest. Regions of interest (ROI) were defined by the AAL ROI archive [64]. To eliminate the asymmetry between the ROI in the left and right hemispheres, ROI was confined to the overlapped region between the original ROI image and its flipped image. The LIs were calculated with the LI toolbox [65], [66]. Negative LI indicated right lateralization; positive LI indicated left lateralization. A one-sample T-test was applied to examine whether LIs in a certain ROI was significantly different from 0. We also measured the reaction time duration of pronunciation and silence interval between syllables for each four-syllable sequence.

### Sound recording analysis

Trials with erroneous pronunciations or naming latency exceeding the range of mean ±2.5 SD were excluded from the analysis. Two participants' data were discarded because of bad recordings and a high error rate. The sound recordings were first epoched trial by trial. Using the software Praat [67], [68] and the program ProsodyPro [28], [69], [70], boundaries between voiced and silent intervals and vocal pulses were marked for each epoch. Manual correction was performed consulting the spectrogram and the sound waveform. The resulting F0 values were smoothed, time-normalized by taking 16 points from each syllable at equal proportional intervals, and speaker-normalized through division by the speakers' F0 ranges (maximum F0 minus minimum F0) after subtracting speakers' mean F0. F0 within a sequence tends to decline over the production, so to reduce this effect, each sequence was detrended and the mean F0 of each syllable was adjusted to 0. We measured the reaction time, duration of the sequences, and the duration of the three silence intervals between the four syllables for each sequence.

## Results

### Behavioral results

**Figure 2:**
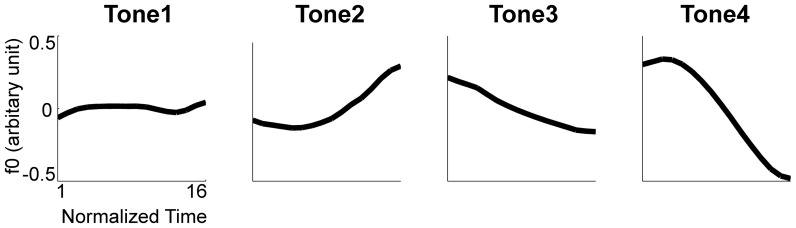
The F0 contours of the four Chinese lexical tones.

**Figure 3:**
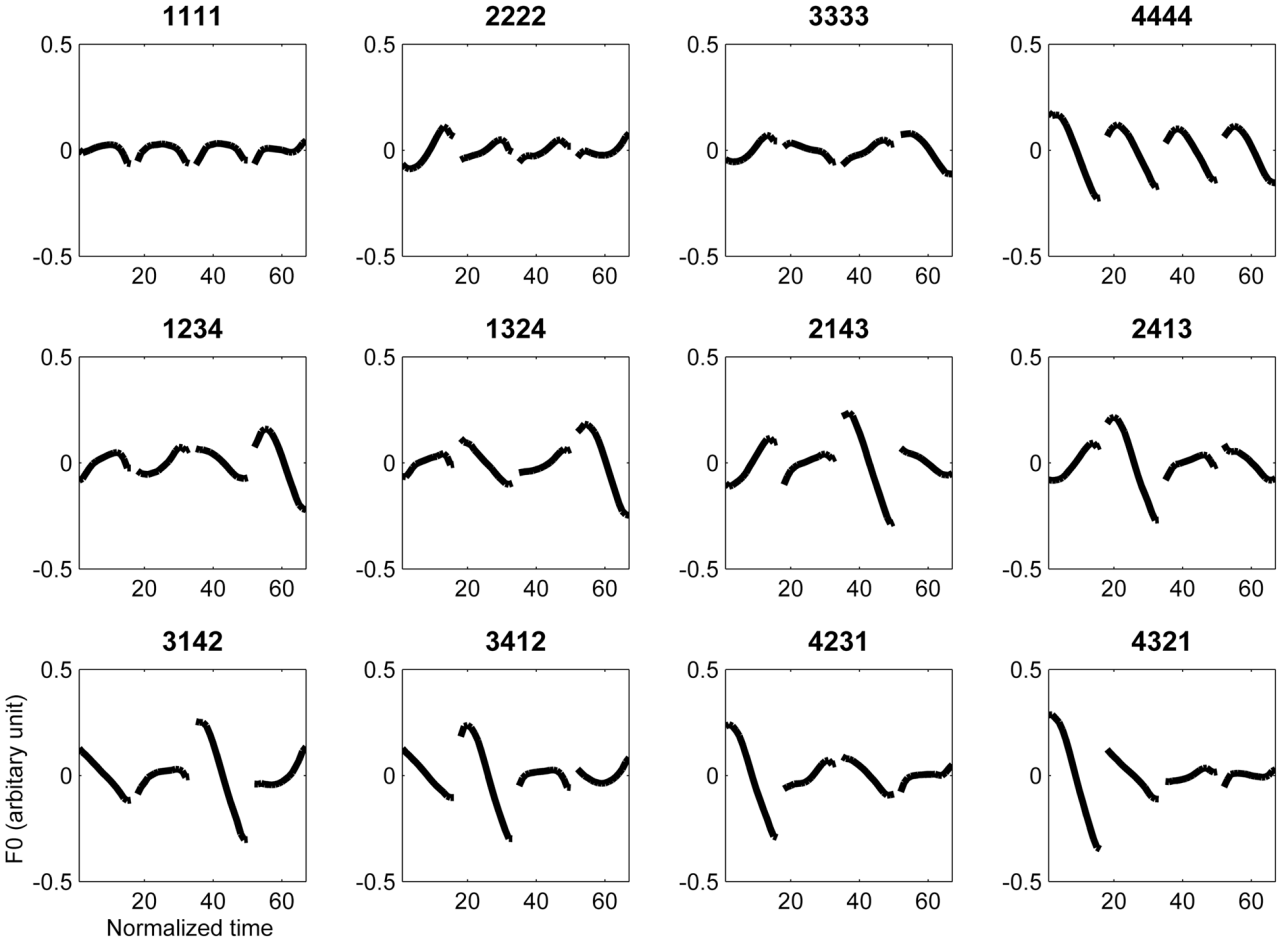
The F0 contours of the twelve tone sequences.

Figure 2 presents the averaged F0 contour of the four tones in monosyllable. The averaged F0 patterns of the 12 sequences are presented in Figure 3.

As clearly shown in Figure 3, the F0 patterns of the first and third Tone 3 in the 3333 sequence deviate from the typical pattern of Tone 3, indicating that the four syllables were treated as two disyllabic chunks and Tone 3 sandhi was applied to the first syllable of each chunk. Namely, sequence 3333 was articulated as 2323 during production. Disyllabic chunking is a natural tendency in Chinese [71]. One-tailed paired T-tests showed that the second silence interval (175 ms) was longer than the first (159 ms; t(12) = 1.94, p<.05) and the third (146 ms; t(12) = 4.30, p<.01) intervals, while the first silence interval was longer than the third (t(12) = 2.36, p<.05).

To quantitatively examine the sandhi Tone 3, the slope of the monosyllable Tone 3, the averaged slope of the first and the third Tone 3 in sequence 3333 (sandhi Tone 3), and the averaged slope of the first and the third Tone 2 in sequence 2222, was analyzed with one-way repeated ANOVA. Their slopes were significantly different (*F(2,24) = 26.98, p<.01*). Post-hoc comparisons revealed that the slopes of sandhi Tone 3 and Tone 2 were not different from each other (*p>.05*), while they both differed from the slope of monosyllable Tone 3 (*p<.01*).

Paired T-tests were performed for the two contrasts in interest—i.e., the mixed sequences vs. baseline and the 3333 vs. baseline—on RT, duration, and error rate. The difference between baseline and mixed sequences was significant for RT (874 ms vs. 1,121 ms; t(12) = −6.65, p<.01), duration (1,321 ms vs. 1,551 ms; t(12) = −3.26, p<.01), but not for error rate (4% vs. 4%; t(12) = 0.04, p>.05). The difference between 3333 and the other baseline was significant for only RT (969 vs. 874; t(12) = 3.83, p<.01), but not for duration (1,292 vs. 1,321; t(12) = −2.10, p>.05) and error rate (3% vs. 4%; t(12) = −1.20, p>.05).

### Imaging results

**Figure 4:**
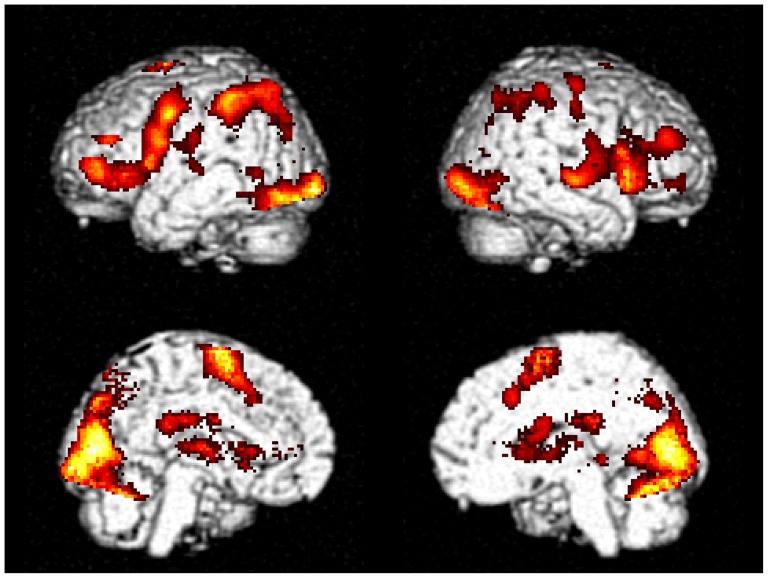
Tone 3 sandhi effect. The activations were thresholded at *p*<0.001 and corrected at cluster level FDR *p*<0.05.

Sequence 3333 showed higher activation in the middle frontal gyrus (MFG), inferior frontal gyrus (IFG), insula, SMA, precentral gyrus, superior temporal gyrus (STG), superior parietal lobule (SPL), inferior parietal lobule (IPL), precuneus, cuneus, fusiform gyrus, lingual gyrus, middle occipital gyrus, thalamus, and caudate (Table 1, Figure 4).

**Table 1:** Tone 3 sandhi effect.

	Left Hemisphere					Right Hemisphere				
	T-value	x	y	z	BA	T-value	x	y	z	BA
MFG	4.23	−30	51	7	10	4.32	42	48	22	10
	3.54	−44	42	20	46					
IFG	4.07	−42	45	1	10	4.11	57	16	14	44
	3.54	−53	11	25	9	3.6	57	16	−1	47
Insula	4.23	−36	23	−1	47	5.74	42	19	−3	47
SMA	6.25	0	3	66	6					
Precentral G	5.56	−50	2	44	6	4.4	46	−13	58	4
STG	3.55	−63	−17	8	42	5.14	63	−4	2	22
SPL	5.41	−26	−66	46	7	3.72	28	−61	55	7
IPL	5.19	−44	−44	50	40	5.55	36	−44	43	40
Precuneus	3.47	−2	−76	42	7					
Cuneus	6.98	−24	−93	−2	18	5.45	2	−77	8	18
Fusiform G	5.81	−42	−71	−12	19					
Lingual G	5.24	−6	−93	−2	17					
Calcarine						4.24	16	−54	6	30
MCG						4.53	26	−84	−9	18
Thalamus	3.39	0	−5	8						
Caudate						4.22	10	12	3	

Note: Thresholded at p<.001 (FDR corrected at cluster level, p<.05). BA = Brodman Area; *x*/*y*/*z* = Talairach-coordinates [81].

**Figure 5:**
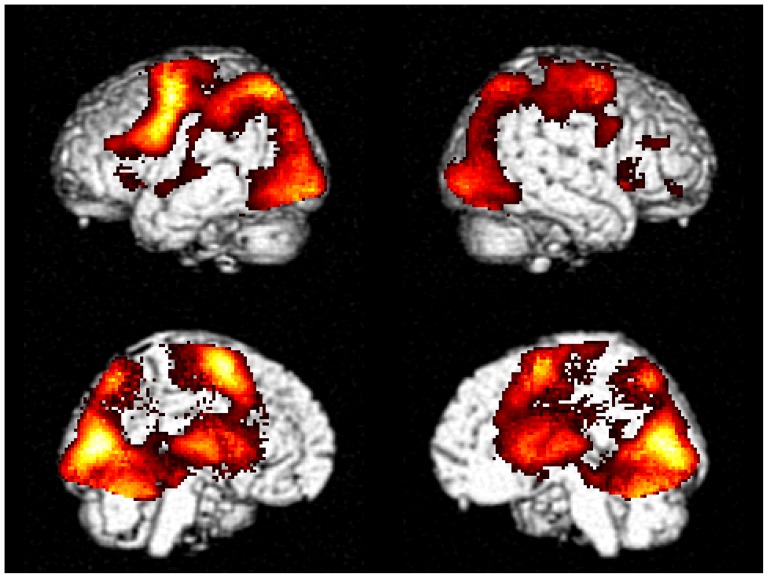
Mixed sequence effect. The activations were thresholded at *p*<0.001 and corrected at cluster level using FDR *p*<0.05.

Mixed sequences elicited higher activation in the bilateral MFG, insula, SMA, medial frontal gyrus, STG, precentral gyrus, postcentral gyrus, SPL, precuneus, cuneus, fusiform gyrus, inferior occipital gyrus (ICG), lingual gyrus, thalamus, putamen, caudate, cerebellum (Table 2, Figure 5).

**Table 2:** Mixed sequence effect.

	Left Hemisphere					Right Hemisphere				
	T-value	x	y	z	BA	T-value	x	y	z	BA
MFG	4.14	−46	34	19	46	7.93	34	3	57	6
Insula	5.23	−32	23	−1	13	5.7	32	19	−1	
SMA	13.75	−2	8	53	6					
MedialFrontal G					3.95	4	−20	69	6	
Precentral G	10.45	−32	1	57	6	5.06	46	−13	56	4
	12	−51	7	31	9	3.54	32	−18	65	6
						4.88	48	9	29	9
Postcentral G	4.2	−61	−16	27	1	7.25	48	−27	44	2
STG	3.96	−63	−17	6	22					
SPL	13.32	−30	−58	53	7	7.32	28	−61	55	7
Precuneus	4.61	0	−67	49	7					
Cuneus	11.98	−12	−73	13	23					
Fusiform G	6.76	−42	−71	−12	19					
ICG	9.58	−30	−91	−2	18					
Lingual G	6.16	−8	−95	−2	17	9.27	4	−85	4	17
						8.7	10	−80	−9	18
						7.04	12	−54	8	30
Thalamus	7	0	−9	10						
Putamen	7.55	−20	6	2						
Caudate	7.24	−12	1	11		7.33	18	1	20	
						6.41	10	4	3	
Cerebellum	7.41	0	−47	−13						

Note: Thresholded at p<.001 (FDR corrected at cluster level, p<.05). BA = Brodman Area; *x*/*y*/*z* = Talairach-coordinates [81].

**Figure 6:**
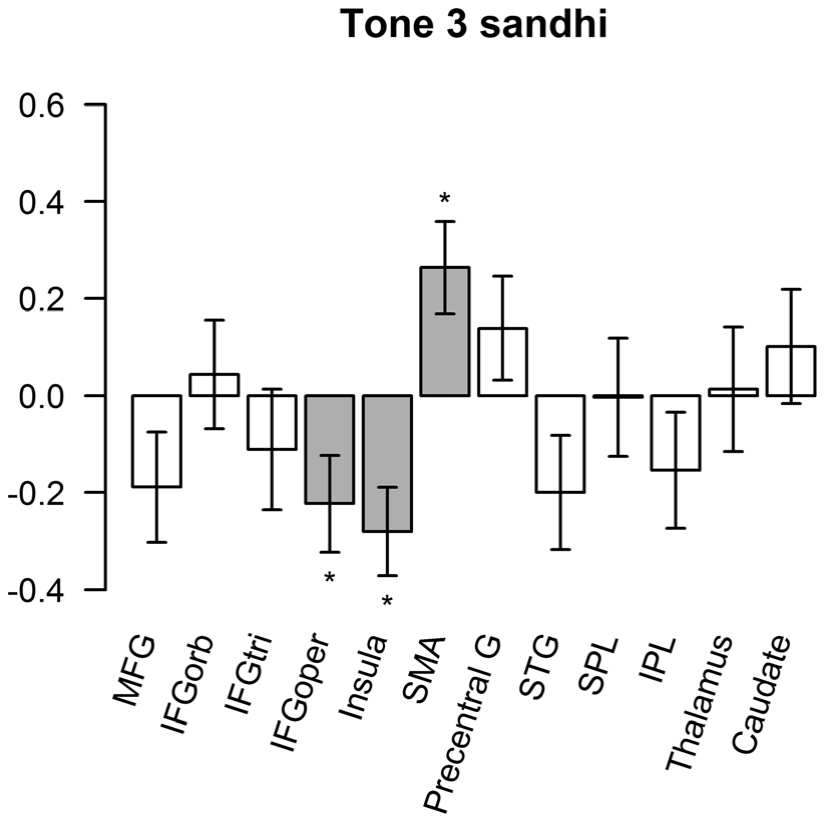
LI in regions showing significant Tone 3 sandhi effect. Error bars represent 1 standard error of mean (SEM) across Participants after subtraction of each participant's individual mean. Bars in grey and star symbols indicate LI significantly different from zero.

**Figure 7:**
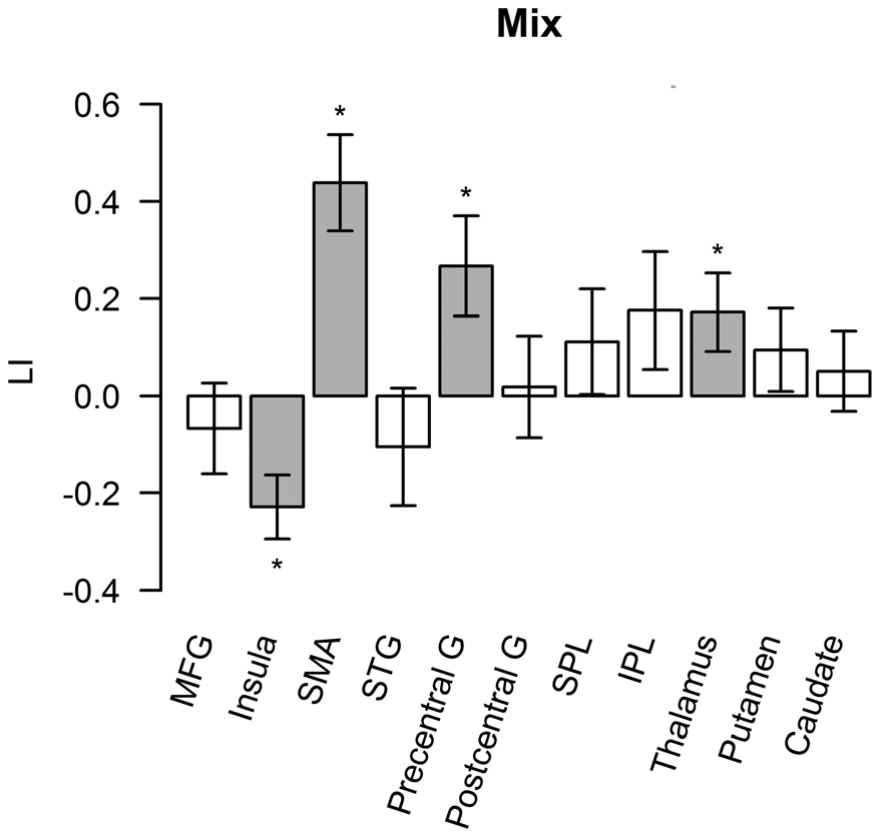
LI in regions showing significant mixed sequence effect. Error bars represent 1 SEM across participants after subtraction of each participant's individual mean. Bars in grey and star symbols indicate LI significantly different from zero.

We calculated the LI in regions that showed significant effects in the two contrasts of interest. As shown in Figure 6, sequence 3333 showed right-lateralized activation in the pars opercularis of IFG (t(20) = −2.25, p<.05) and insula (t(19) = 2.72, p<.05) and left lateralization in SMA (t(19) = 2.72, p<.05). (The degree of freedom was 19 when activation in one of the participants was too weak to measure LI.) Mix sequences showed right-lateralized activation in the insula (t(20) = −3.48, p<.01) and left-lateralized activation in the SMA (t(20) = 4.42, p<.01), precentral gyrus (t(20) = 2.58, p<.05), and thalamus (t(20) = 2.14, p<.05) (Figure 7).

## Discussion

**Figure 8:**
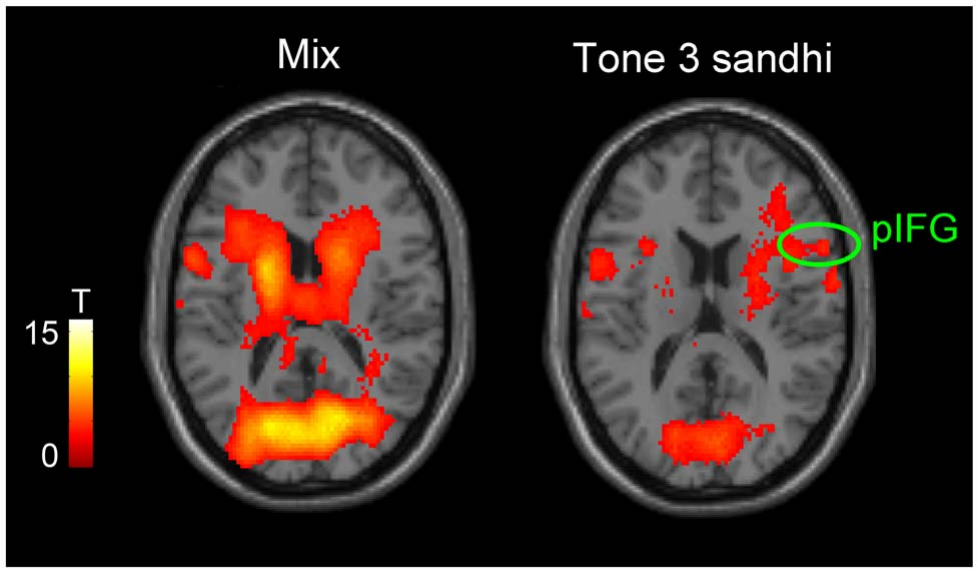
Tone 3 sandhi and mixed sequence effects (Z = 16). The activations were thresholded at *p*<0.001 and corrected at cluster level using FDR *p*<0.05.

This study aims to investigate the neural substrate underlying Tone 3 sandhi and tone sequencing in Mandarin Chinese. Tone 3 sandhi was clearly demonstrated in the F0 contour of sequence 3333. The slope of sandhi Tone 3 deviated from the typical pattern of Tone 3, but was not significantly different from that of Tone 2 (Figure 2 and Figure 3). That is, Tone 3 was substituted with Tone 2. According to current speech production models [42]–[44], the substitution of the articulatory target was predicted to involve left posterior IFG. However, physically, tone production is similar to singing and it has been suggested that in singing, the right posterior IFG might play a role similar to the left posterior IFG in speech production [46], [51]. From our fMRI data, we found that both the sequence 3333 and mixed sequences elicited activation in broadly distributed regions within the speech production network, but the right-lateralized posterior IFG activation was observed for only the sequence 3333 (Figure 4 and Figure 8). The distributed activation pattern lent support to the possibility that sequence 3333 was treated as a mixed sequence—i.e., 2323—in the brain.

Based on our findings, we propose that tones and segments are processed in the left and right hemispheres in parallel, but their integration, or the product of their integration, is hosted in the left hemisphere. Being independent of segments [28], Tone 3 sandhi is believed to be right-lateralized. Previous studies have reported that, compared to untrained English speakers, native Mandarin Chinese speakers [17]–[20] and trained English speakers [21] show more activation in the left hemisphere for tone discrimination. Left lateralization in native and trained speakers may reflect the elevated ability to integrate tone and segment. Note that we did not suggest that the left lateralization of language processing is driven by only the physical properties of speech input/output. What we want to point out is that the physical properties might play a role more important than implied by previous studies.

There are several possible answers to why the parallel processing streams of segment and tone converge at the left hemisphere. One explanation is that, regardless of speech or non-speech, the coordination of complicated movements is processed in the left hemisphere. For example, complex hand movements and coordination of two hands are left-lateralized [72], [73]. Another possibility is that segments are important for word recognition [23]. Since there are only four lexical tones but thirty-one segments in Chinese, the segments are more useful in distinguishing words. However, it is beyond the extent of this study to distinguish between these two possibilities.

We found higher right IFG activation and longer RTs for sequence 3333 in comparison to other repeated sequences, and this is consistent with what we expected. However, one could argue that the findings could possibly result from the inherent difficulty of Tone 3 production, because both children [36], [74] and second language learners [21] of Chinese are reported to commit a significant number of Tone 3 errors. We find this possibility to be unlikely because our participants were native Chinese speakers. Tone error is very rare in adult native speakers [76], [77]. For children and second language learners of Chinese, it can also be a case that Tone 3 sandhi makes the learners confuse Tone 3 with Tone 2 [74], [75]. And, actually, a large proportion of Tone 3 errors were replacement of Tone 3 by Tone 2 [21], [36], [74]. We therefore consider that the right IFG activation for sequence 3333 cannot be exclusively explained by the difficulty account.

Our study points out one missing part—tone processing—in current speech production models. For example, by making analogue between tone and syllable, we can apply the HSFC model to Tone 3 sandhi. That is, Tone 3 sandhi will activate the motor program of Tone 2 in BA 44, which in turn inhibits the auditory representation of Tone 2, preventing the resulted 23 sequences to be detected as error. However, our results reveal that the activation in the posterior IFG is right-lateralized rather than left-lateralized for the tone target, which shows that treating syllables and tone alike is not appropriate. Further, the model doesn't explain how and where the condition to trigger Tone 3 sandhi—i.e. two Tone 3s in one chunk—is processed in the brain, which indicates that more investigation into context-dependent variation is needed [78].

To reveal the brain region responsible for tone sequencing, we contrasted the mixed sequences with the repeated sequences [59], [79]. The mixed sequences (Figure 5 and Table 2), as well as sequence 3333 (Figure 4 and Table 1), elicited larger activation in broadly distributed regions within the speech production network. Our findings suggest that mixed sequences not only involved processing for sequencing, but also increased the loading on target retrieval, motor execution, and feedback monitoring. Similar findings have been reported in a study comparing mixed and repeated syllable sequences—e.g., “ka-ru-ti” vs. “ta-ta-ta” [59]. There are two regions that show significant lateralization in our findings: the left-lateralized SMA and the right-lateralized insula. Activation of the right insula has been observed during overt singing and is related to motor coordination [50]. According to previous electrophysiological [56], [80] and imaging studies [57]–[59], SMA is involved in sequencing. Therefore, the left-lateralized SMA activation may imply a sequencing unit to be a composite of tone and segment—for example, a tonal syllable.

In summary, in this study the repeated and mixed tone sequences were incorporated to examine [the?] neural substrates of lexical tone production. First, the sequence 3333 induced application of Tone 3 sandhi and resulted in a right-lateralized brain activation in the IFG for production. Because Tone 3 sandhi is independent of segments, this finding indicates that the role of physical properties of speech input/output on language lateralization has been underestimated. Second, neural substrates for tone sequencing were revealed as well. Therefore, this study not only helps shed light on the understanding of lexical tone processing, but also points out a missing part of the current speech production models—tone processing.
